# Mosquitocidal Activity and Mode of Action of the Isoxazoline Fluralaner

**DOI:** 10.3390/ijerph14020154

**Published:** 2017-02-06

**Authors:** Shiyao Jiang, Maia Tsikolia, Ulrich R. Bernier, Jeffrey R. Bloomquist

**Affiliations:** 1 Emerging Pathogens Institute, Department of Entomology and Nematology, University of Florida, Gainesville, FL 32610, USA; shiyao.jiang@ufl.edu (S.J.); maia.tsikolia@ars.usda.gov (M.T.); 2 USDA-ARS, Center for Medical, Agricultural, and Veterinary Entomology, Gainesville, FL 32608, USA; uli.bernier@ars.usda.gov

**Keywords:** *Aedes aegypti*, *Anopheles gambiae*, *Drosophila melanogaster*, fipronil, GABA receptor

## Abstract

Mosquitoes, such as *Aedes aegypti* and *Anopheles gambiae*, are important vectors of human diseases. Fluralaner, a recently introduced parasiticide, was evaluated as a mosquitocide in this study. On *Ae. aegypti* and *An. gambiae* fourth-instar larvae, fluralaner had 24-h LC_50_ (lethal concentration for 50% mortality) values of 1.8 ppb and 0.4 ppb, respectively. Following topical application to adult *Ae. aegypti*, fluralaner toxicity reached a plateau in about 3 days, with 1- and 3-day LD_50_ (lethal dose for 50% mortality) values of 1.3 ng/mg and 0.26 ng/mg, suggesting a slowly developing toxicity. Fipronil outperformed fluralaner by up to 100-fold in adult topical, glass contact, and feeding assays on *Ae. aegypti*. These data show that fluralaner does not have exceptional toxicity to mosquitoes in typical exposure paradigms. In electrophysiological recordings on *Drosophila melanogaster* larval central nervous system, the effectiveness of fluralaner for restoring nerve firing after gamma-aminobutyric acid (GABA) treatment, a measure of GABA antagonism, was similar in susceptible Oregon-R and cyclodiene-resistant *rdl*-1675 strains, with EC_50_ (half maximal effective concentration) values of 0.34 µM and 0.29 µM. Although this finding suggests low cross resistance in the presence of *rdl*, the moderate potency, low contact activity, and slow action of fluralaner argue against its use as an adult mosquitocide for vector control.

## 1. Introduction

Gamma-aminobutyric acid (GABA) is present in both mammals and invertebrates and is an important inhibitory neurotransmitter [1]. Among the different types of GABA receptors, the GABA receptor-chloride channel complex is an important target site for insecticides, such as lindane and fipronil [2]. However, target site mutations have significantly reduced the utility of conventional GABAergic insecticides. Mutation A301S [3,4], which was originally described as A302S [5] and studied in cultured neurons from *Drosophila melanogaster*, confers ca. 100-fold and ca. 1000-fold resistance to picrotoxinin and lindane, respectively [6]. Therefore, development of insecticides that work on novel binding sites of this receptor could help avoid cross resistance problems and contribute to effective control of pest insects.

**Figure 1:**
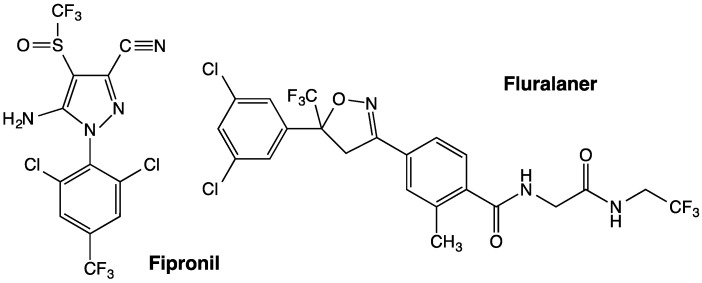
Chemical structures of fipronil and fluralaner.

Isoxazolines and *meta*-diamides have emerged as second-generation GABAergic compounds in the search for novel insecticides [7]. Two representative isoxazolines, fluralaner (Figure 1) and afoxolaner, were developed by scientists from Nissan Chemical Industries in Japan and DuPont in the U.S., respectively [8,9]. Both compounds have been approved by the U.S. Food and Drug Administration (FDA) to be commercialized as parasiticides. Fluralaner shows no cross-resistance in both in vivo and in vitro studies against various insects species compared to other classic GABAergic compounds [10,11], and demonstrates good selectivity towards mammals vs. invertebrate pests [10,12]. According to Gassel et al. [11], fluralaner has toxicity greater than fipronil against six insect and parasite species, and blocks homo-oligomeric GABA receptors expressed in cell lines with high potency. The only available data on mosquito toxicity of fluralaner is that a low concentration of 1.2 ppt (parts per trillion, 10^−12^ g/mL) killed more than 90% of first-instar *Ae. aegypti* larvae, which is ca. 16,000-fold more potent than fipronil [11]. The goal of the present study is to investigate fluralaner toxicity on *Ae. aegypti* and *An. gambiae* via different exposure routes, and its activity on native GABA receptor responses of CNS preparations of susceptible and resistant *D. melanogaster* strains.

## 2. Materials and Methods

### 2.1. Chemicals

Samples of dieldrin and l-aspartic acid were purchased from Sigma-Aldrich Chemical Co. (St. Louis, MO, USA). Fipronil (Figure 1) was donated by Rhône-Poulenc Ag Co. (now Bayer CropScience, Research Triangle Park, NC, USA), and Triton X-100 was acquired from Thermo Fisher Scientific Inc. (Waltham, MA, USA). Diethyl maleate (DEM) was purchased from Sigma-Aldrich Chemical Co., and piperonyl butoxide (PBO) and *S,S,S*-tributyl phosphorotrithioate (DEF) were obtained from Chem Service Inc. (West Chester, PA, USA). Rapeseed-oil methyl ester (RME) was purchased from UCY Energy (Alfter, Germany), and silicon oil was acquired from Dow Corning Co. (Auburn, MI, USA). Ethanol, acetone and dimethyl sulfoxide (DMSO) used as solvents were obtained from Sigma-Aldrich Chemical Co.

Fluralaner was extracted and purified from a commercial canine formulation of BRAVECTO™ Chews (for large dogs, 44–88 lbs, each containing 1 g of fluralaner), manufactured by Merck Animal Health. Isolation of the compound was performed with a Teledyne Isco flash chromatography system (Lincoln, NE, USA) using hexanes/ethyl acetate as eluent system. Solvents, hexanes and ethyl acetate were obtained from Acros Organics (Morris Plains, NJ, USA). Melting point was determined on a hot-stage apparatus and is uncorrected. Nuclear magnetic resonance (NMR) analyses were performed at the Nucleic Magnetic Resonance Facility of the University of Florida. NMR spectra were recorded in CDCl_3_ with tetramethylsilane as the internal standard for ^1^H (500 MHz) and CDCl_3_ as the internal standard for ^13^C (125 MHz).

One half of a Bravecto Chew (3.70 g) was placed in a 250-mL separatory funnel, water (80 mL) was added and the funnel was shaken well to obtain a homogenous suspension. Then, ethyl acetate (80 mL × 3) was used for extraction. Organic phases were combined, washed with aq. Na_2_CO_3_ solution, aq. 1 N HCl solution, brine and then dried over anhydrous sodium sulfate. Any remaining solvent was distilled off under reduced pressure and the resultant residue was purified by flash chromatography using hexanes/ethyl acetate nonlinear gradient to obtain 0.48 g of the target product 4-[(5*RS*)-5-(3,5-dichlorophenyl)-4,5-dihydro-5-(trifluoromethyl)-1,2-oxazol-3-yl]-*N*-[2-oxo-2-(2,2,2-trifluoroethylamino)ethyl]-*o*-toluamide. Product identity and purity (99%) were confirmed with thin layer chromatography (TLC) and NMR analysis. It was a colorless solid after recrystallization from ethanol; m.p. 171.0–172.0 °C; ^1^H-NMR (CDCl_3_) *δ* 7.54–7.41 (m, 6H), 7.29 (t, *J* = 6.5 Hz, 1H), 6.92 (t, *J* = 6.9 Hz, 1H), 4.21 (d, *J* = 5.1 Hz, 2H), 4.10–4.06 (m, 1H), 3.97–3.88 (m, 2H), 3.72–3.68 (m, 1H), 2.44 (s, 3H). ^13^C-NMR (CDCl_3_) *δ* 169.8, 169.4, 155.4, 138.8, 137.4, 137.1, 135.6, 129.8, 129.5, 129.4, 127.7, 123.4 (q, *J* = 278.9 Hz), 123.7 (q, *J* = 285.4 Hz), 87.3 (q, *J* = 30.5 Hz), 43.9, 43.6, 34.9 (q, *J* = 40.7 Hz), 19.7.

### 2.2. Insects

Fourth-instar *Ae. aegypti* larvae were kindly provided by the Center for Medical, Agricultural & Veterinary Entomology (CMAVE), U.S. Department of Agriculture-Agriculture Research Service, Gainesville, FL, USA. The larvae were fed a mixture of ground liver and yeast, maintained under 75% relative humidity and 28 °C, with a 12 h:12 h dark:light cycle, and reared to adulthood for bioassays. Eggs of *An. gambiae* were provided by BEI Resources under the CDC-MR4 program. The emerged larvae were fed with fish flakes (Tetra, Blacksburg, VA, USA), maintained under 75% relative humidity and 28 °C, with a 12 h:12 h dark:light cycle, and reared to adulthood for bioassays. Bioassays were performed on susceptible G3 (MRA-112) strain [13].

Susceptible (Oregon-R) and cyclodiene-resistant (*rdl*-1675) strains of *D. melanogaster* were used in bioassays and electrophysiology experiments. The Oregon-R strain was originally provided by Dr. Doug Knipple from Cornell University, Ithaca, NY, USA, and maintained in culture at the University of Florida since 2009. The *rdl*-1675 strain was purchased from the Bloomington Drosophila Stock Center at Indiana University, Bloomington, IN, USA. Both strains were reared at 21 °C and provided with artificial media purchased from Carolina Biological Supply, Burlington, NC, USA.

### 2.3. Larval Mosquito Bioassays

Compounds were dissolved in ethyl alcohol, followed by serial dilution to generate an appropriate number of concentrations. A 5 µL aliquot (or more depending on compound solubility) of this solution was added to 5 mL of tap water containing 10 fourth-instar larvae of each treatment group. Tap water treated with the corresponding amount of ethanol was used as the control group. Larvae were held at room temperature (21 °C) and observed periodically for behavioral effects, such as convulsions and paralysis, for the first 4 h after treatment. Larval mortality was recorded after 24 h, 48 h and 72 h, or until toxicity reached a stable plateau. In these assays, the plateau was defined as the treatment day where toxicity was not significantly different from the value observed 2 days later. Larvae were fed during the experiment with a few grains of ground fish flakes added to the petri dish every day. A Triton X-100 solution of 10 ppm was also tested to see whether it would increase the toxicity of fluralaner. 

Paralytic activity of compounds to headless larvae was assessed as described by Islam and Bloomquist [14], with slight modification. Heads of fourth-instar larvae were detached with forceps and treated as described above, except in physiological saline instead of tap water. The saline was composed of (mM): NaCl (154), KCl (2.7), CaCl_2_ (1.4), HEPES (4) at pH 7.2. Mosquito saline treated with 0.5% ethanol and 100 ppm l-aspartic acid were used as negative and positive controls, respectively. An insect pin was used to probe larvae every hour to observe swimming behavioral responses. Compared to the control group, larvae showing irregular behaviors such as consistent convulsion, twitching or only slight movement were counted as paralyzed. Each concentration was repeated on at least three different batches of mosquitoes. LC_50_ (lethal concentration for 50% mortality) and PC_50_ (concentration for 50% paralysis) values were calculated as described in the statistics section.

### 2.4. Adult Mosquito Bioassays

For topical toxicity assays [15], 5–7 days old adult female mosquitoes (non-blood fed, *n* = 10) were chilled on ice for 2 min, during which time 0.2 µL of insecticide solution (in ethanol) was applied to the thorax by a hand-held microdispenser (Hamilton, Reno, NV, USA). A solvent-only treatment was included in each experiment as a negative control. Following treatment, mosquitoes were transferred to paper cups covered with netting. A 10% sugar solution in tap water was supplied via a cotton ball placed on the netting and changed every day. Paper cups were held at room temperature (21 °C), and the mosquitoes were observed for behavioral effects such as convulsion or paralysis and the onset of toxicity for the first 4 h. To test for synergistic effects, synergists were applied topically to the abdomen of mosquitoes 4 h before treatments (PBO, 500 ng per mosquito; DEF, 200 ng per mosquito; or DEM, 1 µg per mosquito) at amounts that generally produced little or no mortality. Mortality was recorded after 24 h, 48 h, and 72 h, or until toxicity reached a stable plateau. Each dose was repeated on at least three different batches of mosquitoes. The LD_50_ (lethal dose for 50% mortality) without synergist and LD_50_ with synergist were calculated, as described in the statistics section. The synergist ratio was determined by the equation: LD_50_ of insecticide alone/LD_50_ of insecticide + synergist.

Mosquito injection bioassays [16] were performed under a microscope with a manual microsyringe pump (World Precision Instruments, Sarasota, FL, USA) and a fine glass pipette (TW100-4, World Precision Instruments) fabricated by a pipette puller (Sutter Instrument, Novato, CA, USA), and broken at the tip (ca. 20 µm). Compound solutions were prepared in mosquito saline (described in headless larvae assays) with 5% ethanol as a vehicle. A 5% ethanol solution in saline was used as control. Non-blood fed female mosquitoes (5–7 days post-emergence, *n* = 10) were chilled on ice and injected with 0.2 µL compound solution into the side of their thorax. Treated mosquitoes were then placed into paper cups and maintained as described above for topical assays. Each dose series was repeated on at least three different batches of mosquitoes, with LD_50_ values calculated as described in the statistics section.

For feeding assays [17], female mosquitoes (5–7 days of age and non-blood fed) were starved for 6 h and chilled on ice for 2 min, then transferred to glass test tubes (*n* = 10). Compound solutions were prepared in 10% sugar water with 0.5% ethanol used as vehicle. The control group was assigned 10% sugar water with 0.5% ethanol alone. Cotton balls were treated with 1 mL of prepared compound solution and inserted at the top of the test tubes. Test tubes were maintained at room temperature (21 °C), with the cotton ball changed and solution reapplied every day. To assess synergist effects on feeding assays, synergists (compounds and doses as given above) were topically applied 4 h before treatment. Mortality data was recorded after 24 h, 48 h, and 72 h, or until toxicity reached a stable plateau, with each experiment repeated at least three times. LC_50_ values were calculated as described in the statistics section.

A contact filter paper toxicity assay was conducted according to WHO protocol [18]. Adult female mosquitoes (*n* = 10) were 5–7 days of age and non-blood fed at the time of experimentation. Serial dilutions of each insecticide dissolved in ethanol were prepared prior to treatment and 2 mL of each concentration was applied to a 180 cm^2^ (12 cm × 15 cm) filter paper (Chromatography Paper, Fisher Scientific Pittsburgh, PA, USA). Papers were left to dry for 24 h prior to use. Mosquitoes were chilled for 2 min on ice, after which they were transferred to a WHO cylindrical plastic holding chamber and held for 1 h to acclimatize. The mosquitoes were then gently transferred into the treatment chamber, which contained the treated paper, and exposed for 1 h in the vertical tube. After the 1-h exposure time, the mosquitoes were transferred to the holding chamber as described above, with 10% sugar solution supplied on the netting and changed every day. Holding chambers were left at room temperature (21 °C) after the treatment, and mortality was recorded after 24 h, 48 h, and 72 h, or until toxicity reached a stable plateau. Each experiment was repeated on at least three different batches of mosquitoes. LC_50_ values with and without synergist, as well as synergist ratios were calculated, as described in the statistics section.

Additional surface contact assays were conducted in borosilicate glass test tubes (20 mm × 150 mm) with an inner surface area of ca. 85 cm^2^ (Fisher Brand 14-961-33). Compounds were dissolved in acetone and 250 µL was added to each tube, with 250 µL of acetone as the control group. The test tube was carefully rotated to allow the acetone to evaporate and to distribute the compound evenly inside the tube. Adult female mosquitoes (*n* = 10) 5–7 days from emergence (non-blood fed) were chilled on ice for 2 min and transferred into compound-coated tubes. To ensure parallel comparison to WHO paper assay, in another set of experiments mosquitoes were transferred to clean tubes after 1-h exposure to insecticide-coated glass tubes. A cotton ball with 10% sugar water solution was used to seal test tubes and changed every day to ensure food supply for mosquitoes. Test tubes were maintained at room temperature (21 °C) and mortality data was collected after 24 h, 48 h, and 72 h, or until toxicity reached a stable plateau. Each experiment was repeated on at least three different batches of mosquitoes. LC_50_ values were calculated as described in the statistics section.

### 2.5. Adult D. melanogaster Bioassays

Adult females (*n* = 10) from both strains (CS-OR and *rdl*-1675) that were 5–7 days of age were tested in feeding assays and glass contact assays, and the resistance ratio (LC_50_ *rdl*-1675/LC_50_ CS-OR) was assessed via both routes of exposure. Procedures for both assays were the same as for the mosquito bioassays, except that *D. melanogaster* was anesthetized by a constant flow of CO_2_ for several seconds.

### 2.6. Electrophysiological Recording on D. melanogaster Larval CNS

Electrophysiological recordings from *D. melanogaster* third-instar larval CNS were performed as described previously [19], with slight modification. The CNS was dissected in physiological saline containing (mM) NaCl (157), KCl (3), CaCl_2_ (2), HEPES (4), at pH 7.2, and either left intact or transected posterior to the cerebral lobes to eliminate the blood-brain barrier (BBB) and facilitate penetration of chemicals into the central synapses. A recording suction electrode was pulled with a pipette puller (Sutter Instrument, Novato, CA, USA) and broken at the tip (ca. 40 µm) by fine forceps before being filled with saline. Several peripheral nerve trunks were drawn into the electrode to record nerve activity descending from the CNS.

**Figure 2:**
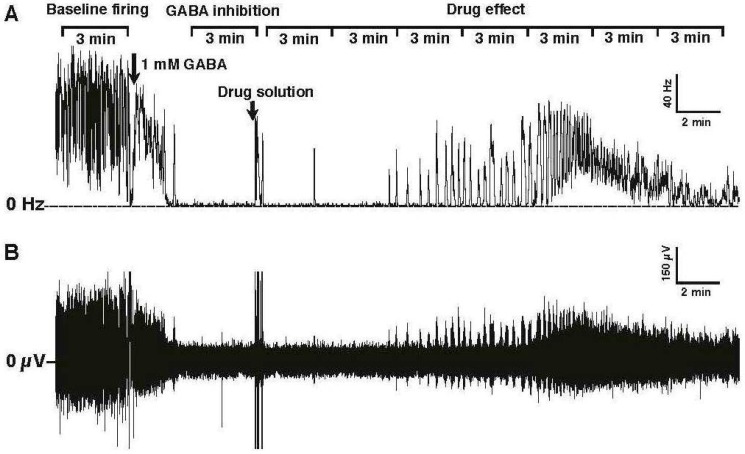
*D. melanogaster* larval CNS recordings and sampling for statistical analysis. (**A**) Nerve firing displayed as a frequency of spikes/s (Hz). For statistical analysis, baseline firing frequency and nerve discharge inhibited by 1 mM GABA were calculated by averaging over 3-min periods as shown in the figure. The drug-induced nerve firing rate was averaged over 3-min periods after treatment; (**B**) Nerve firing of the same preparation displayed as a voltage recording.

Electrical signals were amplified, digitized, and transmitted to the analysis software, wherein spikes were converted to a rate (spikes/s or Hz) by a PowerLab analog to digital converter hardware and LabChart 7 software (ADInstruments, Colorado Springs, CO, USA). To eliminate background noise, spikes were only tallied if they exceeded a fixed threshold, which was set when no peripheral nerves were attached to the suction electrode. After baseline frequency was established, 1 mM GABA was added to the saline bath to inhibit nerve activity, and allowed to incubate for 5 min (Figure 2).

Experimental compounds (e.g., fluralaner) were then added to the saline bath (1 µL of DMSO solution in a 1 mL bath volume) and mixed by gentle pipetting to assess their ability to reverse the inhibitory effect of GABA. The baseline (pre-treatment) and GABA inhibited nerve firing rates were each averaged over a 3 min period. The drug-induced nerve firing rate was averaged over 3 min periods after treatment (Figure 2). Fipronil and dieldrin at a concentration of 10 µM were used as positive controls. If, after GABA treatment, the nerve firing rate recovered to at least 25% of the original pretreatment level, the toxicant was considered to have induced a positive response.

Drug effects on larval CNS with intact BBB also were assessed, with experimental compounds added to the bath after establishing the baseline frequency, and each drug concentration was replicated 3–9 times. Fipronil at 10 µM was used as positive control. Recording data obtained within 36 min after addition of experimental compounds were included in the analysis.

### 2.7. Test of GABAergic Compounds on Mammalian GABA_A_ Receptors

Effects of fluralaner, fipronil, dieldrin, and picrotoxin were tested on GABA α_1_β_3_γ_2_ ion channels expressed in HEK293 cells by ChanTest Corporation, Cleveland, OH, USA. Briefly, HEK293 cells were transfected with cDNA of GABA α_1_β_3_γ_2_ ion channels and maintained in Dulbecco’s Modified Eagle Medium/Nutrient Mixture F-12 with addition of 10% fetal bovine serum, 100 U/mL penicillin G sodium, 100 μg/mL streptomycin sulfate, and 500 μg/mL G418. Extracellular buffer containing (mM): NaCl (137), KCl (4), CaCl_2_ (1.8), MgCl_2_ (1), HEPES (10), Glucose (10), at pH 7.4 was loaded into planar patch clamp (PPC) plate wells (11 µL per well). Cell suspension was then pipetted into the intracellular compartment (9 µL per well) of the PPC planar electrode. With whole-cell recording, effects of compounds on transfected HEK293 cells were detected in agonist mode (application of test compounds only) and in antagonist mode (application of GABA with test compound), with solution added 10 µL/s for 2 s. GABA and picrotoxin were used as agonist positive control and antagonist positive control, respectively.

The agonist effect of the test compounds and GABA (positive control) was calculated as: % activation = (I_TA_/I_Max_) × 100%, in which I_TA_ was the compound-induced current at various concentrations, and I_Max_ was the mean current induced with 300 μM GABA. Inhibitory effect of the test compounds and picrotoxin on the channel was calculated as: % Inhibition = (I_TA_/I_EC80_) × 100%, where I_TA_ was the GABA EC_80_-induced current in the presence of various concentrations of the test compound and I_EC80_ was the mean current elicited with GABA EC_80_. GABA EC_80_ values were selected based on ChanTest historical data: 60 μM for α_1_β_3_γ_2_ GABA ion channels.

Inhibitory concentration-response data were fitted to an equation of the form: % Inhibition = % VC + {(% PC − % VC)/[1 + ([Test]/IC_50_)^N^]}, where [Test] was the concentration of the compound, IC_50_ was the concentration of the compound producing 50% inhibition, N was the Hill coefficient, % VC was the mean current at the passive control (picrotoxin EC_50_), % VC was the mean current at the vehicle control (GABA EC_50_) and % inhibition was the percentage of ion channel current inhibited at each concentration of the test compound. Nonlinear least squares fits were solved with the XLfit add-in for Excel software (Microsoft, Redmond, WA, USA).

### 2.8. Statistical Analysis

Control mortality was corrected by Abbott’s formula and LC_50_, LD_50_, and PC_50_ values were calculated by SAS software (Proc Probit, SAS 9.4, SAS Institute Inc., Cary, NC, USA). EC_50_ and IC_50_ values, and concentration-response curves of drugs in *Drosophila* larval CNS recordings were obtained by nonlinear regression to a four-parameter logistic equation in GraphPad Prism 4.0 software (GraphPad Software, Inc., San Diego, CA, USA). The two-tailed Student’s *t*-test, one-way ANOVA with Bonferroni or Tukey’s post-test used to analyze results between treatment group means, with a *p* < 0.05 considered to be statistically significant.

## 3. Results

### 3.1. Larval Mosquito Bioassays

The 24-h LC_50_ values of fluralaner on fourth-instar *Ae. aegypti* and *An. gambiae* larvae were 1.8 ppb and 0.4 ppb, respectively; a statistically significant difference (*p* < 0.05) by a factor of 4.5 (Table 1). The toxicity of fluralaner on *Ae. aegypti* larvae also increased significantly (*p* < 0.05) by a factor of 3.6 after 48 h of exposure (Table 1).

*Aedes* larval assays performed in mosquito physiological saline and in 10 ppm Triton X-100 solution displayed a small, but statistically-significant increase (*p* < 0.05) of LC_50_ values to 2.9 ppb and 2.7 ppb, respectively, compared to those performed in tap water (Table 1). The 24 and 48 h LC_50_ values of fipronil on fourth-instar *Ae. aegypti* larvae were 23 ppb and 5.1 ppb, respectively, and showed 10- to 13-fold less potency compared to the toxicity of fluralaner in the same assay. The LC_90_ values at the 48-h time point of fluralaner and fipronil on fourth-instar *Ae. aegypti* larvae were 2.2 ppb (1.4–6.0 ppb) and 11 ppb (8–21 ppb), respectively. Paralysis studies with fluralaner on headless and intact *Ae. aegypti* larvae yielded a significant (*p* < 0.05) 10-fold difference in PC_50_ values (Table 1). Intoxication signs of fluralaner in mosquito larvae were spontaneous asynchronous contractions and convulsions, similar to signs of fipronil exposure.

### 3.2. Adult Mosquito Bioassays

**Figure 3:**
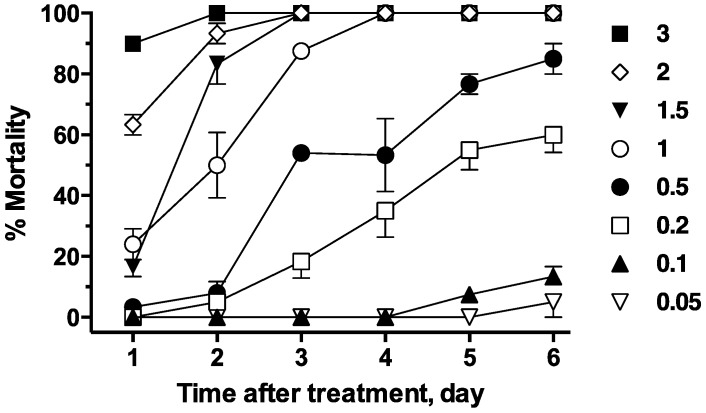
Mortality data for adult topical assay showing the increase of toxicity over time at a range of doses labeled to the right of the symbols and expressed as ng/mosquito. No mortality was observed in controls over six days when treated with solvent vehicle.

In adult mosquito bioassays, intoxication signs included an inability to stand upright, and flying along the bottom of holding container while on their backs. In topical assays, the 24 h LD_50_ values of fluralaner on *Ae. aegypti* and *An. gambiae* were 1.3 ng/mg and 0.29 ng/mg, respectively (Table 2), a species difference of 4-fold. In time course studies with *Ae. aegypti*, the toxicity of fluralaner on mosquitoes increased progressively (Figure 3), and the LD_50_ value following a single topical application decreased significantly (*p* < 0.05; one-way ANOVA, Tukey’s test) on a daily basis until day 3 (Table 2). At day 3, the LD_50_ value was 0.26 ng/mg (Table 2), five-fold lower than that observed on day 1. At observation days 4, 5 and 6, the toxicity did not increase further (*p* > 0.05) compared to the LD_50_ on day 3 (Figure 3), and at the two lowest doses tested did not reach 100% mortality, even after 7 days of observation (Figure 3). For *An. gambiae*, fluralaner toxicity reached its plateau at day 2, with an LD_50_ value of 0.21 ng/mg (Table 2), a difference of only 1.4-fold compared to 24 h. For comparison, topical assays of fipronil on *Ae. aegypti* found an LD_50_ value after 24 h of 0.062 ng/mg (Table 2). Toxicity of fipronil in *Ae. aegypti* topical assay stopped increasing significantly at day 2, with a LD_50_ value of 0.021 ng/mg (Table 2). Fipronil was more active than fluralaner on *Ae. aegypti* in topical assays by 21-fold at 1 day post-exposure and 17-fold at day 2.

For injection bioassays of fluralaner on *Ae. aegypti* adult females, the 24-h LD_50_ value was 0.6 ng/mg (Table 3), with no increase of toxicity at the 48-h and 72-h time point. This LD_50_ value was less than that in 24-h topical assays by a factor of 2. The 24-h LC_50_ values for glass contact and feeding assays were 13 ng/cm^2^ and 49 ppm, respectively (Table 3). Toxicity of fluralaner by feeding and glass contact methods increased by factors of 2- to 4-fold after 48 h, compared to 24-h exposures. For comparison, fipronil had a glass contact LC_50_ value of 10 ng/cm^2^ after 24 h and 0.7 ng/cm^2^ after 48 h, which is a 14-fold increase in toxicity (Table 3).

In feeding assays, fipronil had LC_50_ values of 0.47 ppm and 0.12 ppm for 24 h and 48 h, respectively, and the toxicity did not increase significantly after 48 h (Table 3). Compared to fluralaner, fipronil had ca. 8-fold greater toxicity in glass contact assays at the 48-h time point and ca. 100-fold higher toxicity in feeding assays at 24-h and 48-h time points. At the 24-h time point in the glass contact assay, the difference in toxicity of fluralaner and fipronil was not statistically significant.

In WHO paper assays, fluralaner was dissolved in ethanol, with 3.6 mg/cm² of either silicon oil or RME added to the solvent. These formulations at 2 mg/paper (ca. 11,000 ng/cm^2^) of fluralaner killed at most 12% at the 48-h time point. In glass contact assays with exposure time of 1 h to match the WHO paper assay, fluralaner displayed a LC_50_ value of 504 (152–1338) ng/cm^2^, which is significantly more toxic than that in WHO paper assays by a factor of at least 22-fold.

Synergist assays, in which PBO, DEF, or DEM was topically applied to adult female *Ae. aegypti* 4 h before treatment, were performed in topical and feeding assay formats (Table 4 and Table 5). PBO displayed little effect on fluralaner toxicity in either assay, with synergist ratios of at most 2.2-fold. The synergist DEM decreased toxicity to fluralaner in the feeding assay (Table 4), but increased toxicity up to about 2-fold in topical treatments (Table 5). In contrast, DEF displayed strong synergism of fluralaner in feeding assays (Table 4), but weak synergism in topical assays (Table 5).

### 3.3. Adult D. melanogaster Bioassays

To confirm resistance in the *rdl*-1675 strain, dieldrin feeding assays were performed. In these studies, the susceptible strain (CS-OR) had a LC_50_ value of 2.5 ppm (95% CI = 2.1–3.0 ppm) to dieldrin, whereas the resistant strain (*rdl*-1675) had no mortality when fed 100 ppm dieldrin, indicating a resistance ratio greater than 40-fold. In both glass contact and feeding assays, fluralaner displayed equivalent toxicity to susceptible and resistant strains of *D. melanogaster* (Table 6). The LC_50_ values of both *D. melanogaster* strains were not significantly different from each other at the 24-h and 48-h time point (Table 6). In glass contact assays, LC_50_ values of fluralaner on *Ae. aegypti* and *D. melanogaster* were not significantly different from each other at recorded time points (Table 3 and Table 6). However, the toxicity of fluralaner in feeding assays against *D. melanogaster* was greater than that against *Ae. aegypti* by a factor of ca. seven (Table 3 and Table 6). As was observed for mosquitoes, mortality in *D. melanogaster* increased significantly at 48 h compared to 24 h (Table 6).

### 3.4. CNS Recordings on D. melanogaster

**Figure 4:**
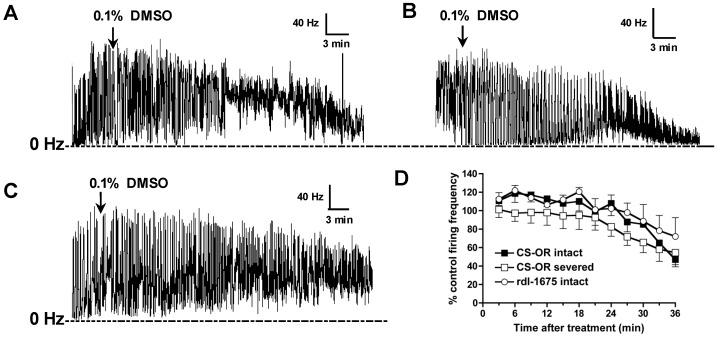
DMSO effects on *D. melanogaster* larval CNS. Ordinate in all traces is spike rate in Hz (spikes/s) vs. time (abscissa). (**A**) Recording of intact CS-OR larval CNS treated with DMSO; (**B**) Recording of severed CS-OR larval CNS treated with DMSO; (**C**) Recording of intact *rdl*-1675 larval CNS treated with DMSO; (**D**) Summary plot of DMSO effects on *D. melanogaster* larval CNS. Each data point was averaged over at least three replicates and error bars represent ± SEM. Dashed line in **A**–**C** is the zero Hz baseline in this and subsequent figures displaying nerve firing measurements.

Initial experiments compared the solvent responses of intact and severed (BBB disrupted) larval CNS preparations. Intact and severed susceptible (CS-OR) CNS treated with DMSO showed a consistent nerve firing pattern, which is cyclic oscillations (Figure 4A–C), but overall spike rates declined over the 30-min observation period (Figure 4D).

On severed CS-OR larval CNS, discharge rates dropped to 72% ± 11% of baseline firing rate 30 min after the addition of DMSO. Firing frequencies of intact CS-OR and *rdl*-1675 (resistant) CNS at 30 min after DMSO addition were 85% ± 3% and 89% ± 18%, respectively, which were greater than that of severed CS-OR CNS (Figure 4D). This difference, however, was not statistically significant.

**Figure 5:**
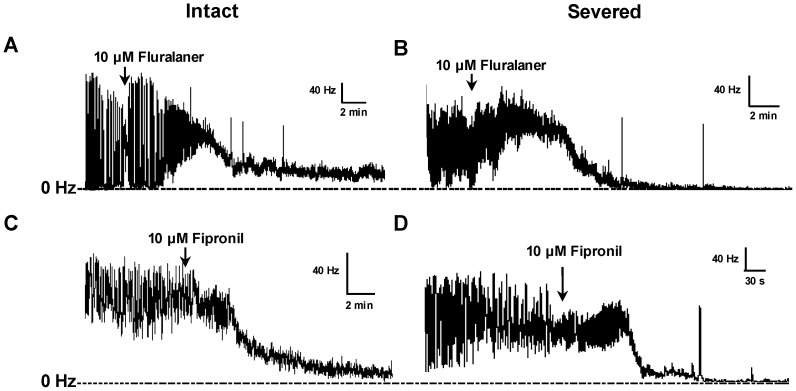
Electrophysiological recordings on intact or severed CS-OR *D. melanogaster* larval CNS. (**A**) Intact CNS treated with 10 µM fluralaner; (**B**) Severed CNS treated with 10 µM fluralaner; (**C**) Intact CNS treated with 10 µM fipronil; (**D**) Severed CNS treated with 10 µM fipronil.

On CS-OR larval CNS preparations, 10 µM fluralaner showed neuroexcitatory effects within five minutes of addition to the bath (Figure 5A,B). After the treatment of 10 µM fluralaner, firing patterns on larval CNS displayed more rapid cyclic oscillations on top of a greater tonic discharge rate, consistent with a disruption of normal pattern generation. Blocking of nerve discharge was observed following the neuro-excitation, and showed some evidence of greater effect in the severed preparations; viz., there is more residual firing evident in the intact preparations. The effects of 10 µM fipronil on intact and severed CN-OR larval CNS were similar to that of 10 µM fluralaner; an excitatory action followed by inhibition of nerve firing (Figure 5C,D).

**Figure 6:**
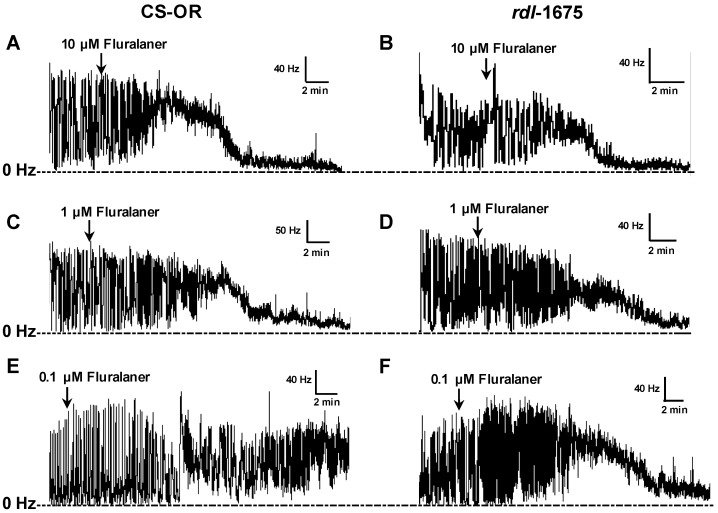
Electrophysiological recordings on CS-OR or *rdl*-1675 *D. melanogaster* intact larval CNS. (**A**) CS-OR CNS treated with 10 µM fluralaner; (**B**) *rdl*-1675 CNS treated with 10 µM fluralaner; (**C**) CS-OR CNS treated with 1 µM fluralaner; (**D**) *rdl*-1675 CNS treated with 1 µM fluralaner; (**E**) CS-OR CNS treated with 0.1 µM fluralaner; (**F**) *rdl*-1675 CNS treated with 0.1 µM fluralaner.

**Figure 7:**
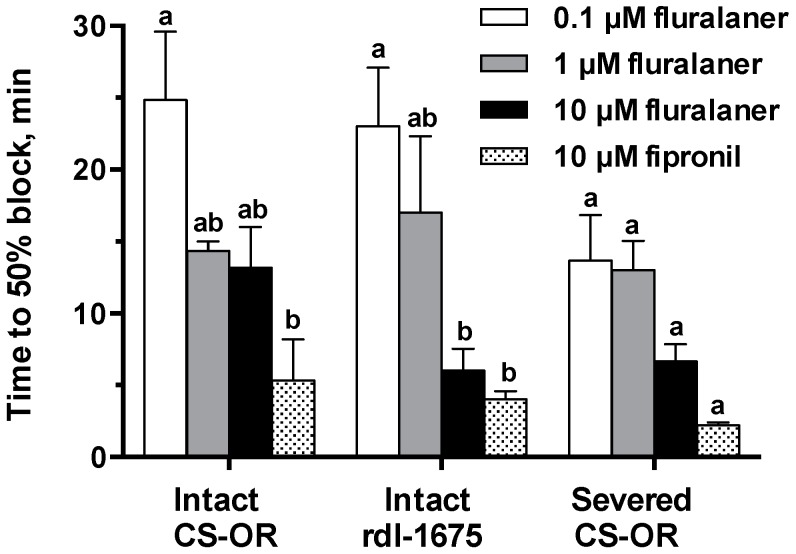
Time to 50% reduction of firing rate after chemical treatments of *D. melanogaster* larval CNS preparations. Each bar is the average of at least three replicates and error bars show ± SEM. Letters indicate statistical significance at the *p* = 0.05 level using a two-way ANOVA calculated with Prism software. Comparisons were made across different treatments within a nerve preparation (*p* < 0.0001) as well as for a given treatment across the different CNS dissections (*p* = 0.1263). Bars not labeled by the same letter are significantly different.

Excitation and nerve blocking effects of fluralaner were concentration-dependent, and lower concentrations required longer incubation times to cause either effect. This relationship is evident in electrophysiological traces (Figure 6), and explored in measurements of time to 50% block of nerve firing (Figure 7). In both susceptible and resistant CNS preparations, reduction in firing occurs more quickly and is more extensive at higher concentrations (Figure 6). Similar results were obtained on intact *rdl*-1675 larval CNS, where 10 µM fluralaner and fipronil blocked nerve discharge significantly faster than 0.1 µM fluralaner (Figure 6 and Figure 7). Time to block of nerve firing was used to quantify effects on the CNS. However, there was not a statistically significant difference in time to 50% nerve block for fluralaner between intact and severed CNS preparations from the susceptible Oregon-R strain (Figure 7). Given the variability inherent to spontaneous nerve discharge recordings, no other analyses were attempted.

**Figure 8:**
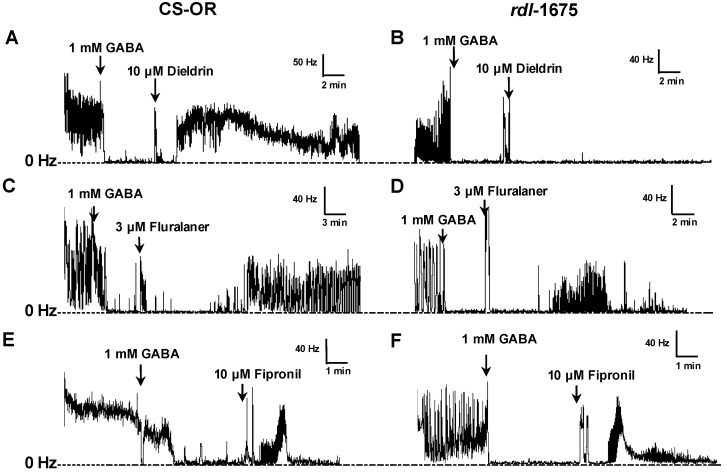
Electrophysiological recordings on severed *D. melanogaster* larval CNS, with GABA inhibition. Test compounds were added after 5-min incubation with 1 mM GABA. (**A**) CS-OR CNS treated with 10 µM dieldrin; (**B**) *rdl*-1675 CNS treated with 10 µM dieldrin; (**C**) CS-OR CNS treated with 3 µM fluralaner; (**D**) *rdl*-1675 CNS treated with 3 µM fluralaner; (**E**) CS-OR CNS treated with 10 µM fipronil; (**F**) *rdl*-1675 CNS treated with 10 µM fipronil.

**Figure 9:**
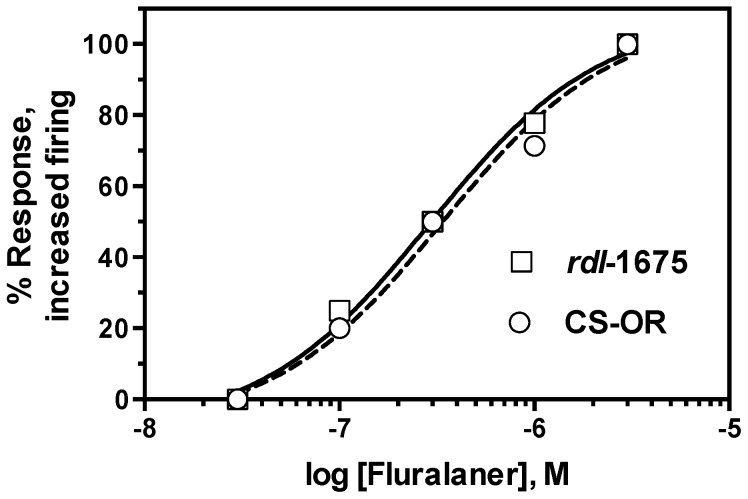
Concentration response curve (% responding preparations) of fluralaner on resistant and susceptible strains of *D. melanogaster*, in transected larval CNS recordings. Fluralaner was added after a 5-min incubation of 1 mM GABA to inhibit nerve activity. Solid line and dotted line represent curves for *rdl*-1675 and CS-OR preparations, respectively.

For comparison, dieldrin, fipronil, and fluralaner were applied to severed susceptible and resistant CNS preparations pretreated with 1 mM GABA to inhibit nerve discharge. Dieldrin (10 µM) stimulated recovery of firing in CS-OR CNS (Figure 8A), but on *rdl*-1675 CNS yielded virtually no recovery of nerve activity (Figure 8B). Application of 0.1% DMSO had no effect on GABA-treated CNS preparations (data not shown). At 3 µM, all preparations from both strains showed a positive response (recovery to at least 25% of baseline firing frequency within a 30 min observation period) to fluralaner (Figure 8C,D). Similarly, fipronil at 10 µM showed similar recovery of firing on severed larval CNS in both susceptible and resistant strains (Figure 8E,F). There was no response to 30 nM fluralaner for either *Drosophila* strain, but higher concentrations were effective (Figure 9). For both susceptible and cyclodiene-resistant CNS preparations, 100 nM fluralaner was the lowest concentration that reversed, even partially, the GABA inhibitory effect (Figure 9). The EC_50_ values for fluralaner on CS-OR and *rdl*-1675 severed CNS preparations were 0.34 µM (95% CI: 0.06–1.8 µM) and 0.29 µM (95% CI: 0.09–0.92 µM); not significantly different from each other.

### 3.5. Potency on Mammalian GABA Receptors

Fluralaner was screened on a mammalian GABA_A_ receptor construct under contact by ChanTest^TM^. Only GABA displayed agonist activity among the compounds tested, and it had an EC_50_ value of 11 µM on mammalian GABA_A_ α_1_β_3_γ_2_ receptors. Fluralaner and dieldrin showed low potency for block of these receptors, with IC_50_ values above 30 µM. For comparison, fipronil and picrotoxin had IC_50_ values of 4.9 µM and 3.9 µM, respectively.

## 4. Discussion

Gassel et al. [11] performed toxicity comparisons of fluralaner with other commercialized compounds, including fipronil. On *Ctenocephalides felis* (cat flea), *Ae. aegypti*, *Lucilia cuprina* Meigen (sheep blowfly), and *Stomoxys calcitrans* Linnaeus (stable fly), fluralaner outperformed dieldrin and imidacloprid, as well as deltamethrin, except on *S. calcitrans*. Ozoe et al. [10] also reported that fipronil outperformed fluralaner on *C. felis* by a factor of five in dry film contact assays. For mosquitoes, Gassel et al. [11] reported a single finding that fluralaner (48 h LC_90_ value = 1.2 ppt) was ca. 16,000-fold more potent than fipronil on *Ae. aegypti* first-instar larvae (48 h LC_90_ value = 20 ppb). In the present study, fluralaner gave a 48 h LC_90_ value of 2.2 ppb on fourth-instar *Ae. aegypti* larvae, differing from the data on first-instar larvae by over 1800-fold. However, fipronil showed a 48-h LC_90_ value of 11 ppb, similar to toxicity observed by Gassel et al. [11] for first instars (20 ppb for >90% mortality). In addition, on fourth-instar *Ae. aegypti* larvae, fipronil was 10- to 13-fold less potent than fluralaner. These data suggest that fluralaner might be an excellent larvicide, although the life stage of the mosquito larvae has a large impact on chemical sensitivity. On adult *Ae. aegypti*, fipronil had higher toxicity than fluralaner in topical (7- to 21-fold), feeding (ca. 100-fold), and glass contact assays (8-fold at 48 h). These findings, plus the greater speed of action, suggest fipronil would be a better overall adult mosquitocide, at least in the absence of resistance.

Bioassays and CNS recordings provide some insight into the slow toxicity of fluralaner. It took 3 days for fluralaner toxicity to plateau, and its toxicity was not enhanced much by injection (about two-fold compared to topical application). In contrast, the LD_50_ value by injection can be reduced by more than 10-fold compared to topical treatments for carbamates such as propoxur [16], another compound that must reach central synapses to exert its effects. Cuticle thickness is known to have a significant and positive correlation with the time to knock down by permethrin, suggesting that thicker cuticle led to a slower rate of insecticide penetration [20]. For larval assays of *Ae. aegypti*, the concentration leading to 50% paralysis on intact larvae was 10-fold higher than that for headless larvae. Thus, the cuticle of fourth-instar *Ae. aegypti* proved to be a more important factor influencing fluralaner toxicity than in adults. The large size (molecular weight = 556) and high lipophilicity (log *p* = 5.0) could influence fluralaner penetration of barriers and contribute to its slowly developing toxicity. These barriers would include the blood-brain-barrier, and although there was no significant difference between speed of nerve discharge block in severed vs. intact *D. melanogaster* larval CNS, the blood brain barrier in adult mosquitoes may have a different permeability to fluralaner.

At present, there is no published information on the metabolism of fluralaner in insects. A decrease in toxicity was observed with DEM in adult sugar feeding assays (Table 5), but the overall effect was small, as was the potentiation of toxicity by PBO. Both findings argue against significant glutathione-*S*-transferase and P450 monooxygenase metabolism. DEF is a potent carboxylesterase inhibitor [21] and in this study had the most significant positive synergist ratios in feeding assays with *Ae. aegypti* adults. However, little synergism was noted in topical applications of fluralaner, suggesting that the factor responsible resides in the alimentary system. No ester linkage is present in the fluralaner molecule, but it does have two adjacent amide groups. Carboxylamidases have been identified in different insect species, including *Lepidoptera*, *Orthoptera*, and *Dictyoptera*, which can use *p*-nitroacetanilide as a model substrate, and were found most abundantly in the midgut [22,23]. The only purified carboxylamidase studied from insects was insensitive to DEF [22]; however, a carboxylamidase might be present in adult *Ae. aegypti* that metabolizes fluralaner in a DEF-sensitive fashion. Alternatively, DEF might have other mechanisms for potentiating the oral toxicity of fluralaner. All these possibilities await further investigation.

Fluralaner showed no target site cross resistance with other GABA receptor-directed compounds. Ozoe et al. [10] reported fluralaner to be equally effective in topical assays on dieldrin-resistant and susceptible strains of houseflies, with LD_50_ values of 1.01 ng/mg and 0.85 ng/mg, respectively. These values were similar on a per mg insect weight basis to the topical toxicity found for fluralaner on *Ae. aegypti* adults (1.3 ng/mg at 24 h). Additionally, Asahi et al. [24] showed that fluralaner had similar potent toxicity on fipronil-resistant and susceptible strains of *Laodelphax striatellus* Fallén, whereas fipronil showed a resistance ratio of about 1700. These findings are in agreement with results of both feeding and glass contact assays on dieldrin-resistant and susceptible strains of *D. melanogaster* reported here, where fluralaner showed similar activity at all recorded time points.

On homo-oligomeric RDL (resistant to dieldrin) GABA receptors, fluralaner showed no cross-resistance to classical GABA non-competitive antagonists. Gassel et al. [11] reported fluralaner to be a potent blocker of dieldrin-resistant *D. melanogaster* and *C. felis* RDL recombinant, homomultimeric receptors in cell-based fluorescence dye assays, with IC_50_ values as low as 2.8 nM and 1.7 nM, respectively. In two-electrode voltage clamp (TEVC) recordings of oocytes expressing *M. domestica* RDL receptors, fluralaner had similar IC_50_ values of 2.8 nM on *rdl*-type receptors with A299S mutation and 5.3 nM on wild-type receptors [10]. Fluralaner also had an IC_50_ value of 12 nM on fipronil-resistant RDL receptors from a plant-feeding mite, *Tetranychus urticae* Koch, in TEVC experiments, whereas 30 µM of fipronil only blocked 27% of GABA-induced current [24]. In the present study, fluralaner was tested on native *D. melanogaster* GABA receptors, in situ, instead of heterologously expressed RDL receptors. In line with data on expressed RDL receptors, fluralaner showed similar potent activity on native dieldrin-resistant and -susceptible GABA receptors of *D. melanogaster* larval CNS, with EC_50_ values of 0.29 µM and 0.34 µM, respectively. The lower potency in CNS preparations can be attributed to the need for penetration into the neuropile, as well as any differences between native and recombinant homomultimeric receptors. These results further document that fluralaner has potent activity on insect strains that are resistant to classical GABAergic compounds. 

Gassel et al. [11] also reported 18-fold resistance to fipronil in *D. melanogaster* homo-oligomeric RDL GABA receptors (Ser isoform). In the present study, no resistance to fipronil was observed in RDL larval CNS preparations, at least when tested at 10 µM (Figure 8). We would note that this concentration is over 10-fold greater than the IC_50_ for blocking the Ala isoform in homo-oligomeric receptors, reported to be 663 nM [11]. So, the resistance may be largely circumvented at this concentration.

Good selectivity of fluralaner between mammals and invertebrates also has been demonstrated in both in vivo and in vitro studies. Currently commercialized as a parasiticide, fluralaner was reported to be safe to dogs at or above recommended treatments [25,26]. In radioligand binding studies on rat brain membranes, 10 μM of fluralaner showed around 40% inhibition of radiolabeled 4-ethynyl-4-*N*-propylbicycloorthobenzoate (EBOB) binding, which is more than 2000-fold less sensitive than its binding to housefly GABA receptors [10,12]. Additionally, fluralaner had low activities on either recombinant β_3_ homopentamers or α_1_β_2_γ_2_ heteropentamers [10,12]. In the present study, mammalian GABA_A_ α_1_β_3_γ_2_ receptors were tested against fluralaner and fipronil. In line with previous reports, the IC_50_ value of fluralaner was higher than 30 μM. Moreover, the IC_50_ value of fipronil was 4.9 μM, suggesting fluralaner has a lower toxicity to mammals than fipronil.

The mode of action and toxicology of isoxazolines such as fluralaner are similar to another new insecticide class, the *meta*-diamides, which also work on invertebrate GABA receptors. According to Nakao et al. [27], *meta*-diamide **7** has potent activity on three mutant GABA receptors that are resistant to GABA receptor-directed non-competitive antagonists. Additionally, mutations G336M in M3, I277F and L281C in M1 reduce the activity of fluralaner on RDL GABA receptors, while having only a small impact on the activity of fipronil. Further molecular modeling suggests that *meta*-diamide binds to T9’ to S15’ region in M2, close to the avermectin target site and different from the classical convulsant site [28]. Homology modeling also suggests that fluralaner might share the same binding site as *meta*-diamide **7** [28]. According to most recent Mode of Action Classification Scheme from the Insecticide Resistance Action Committee (IRAC) [2], GABAergic insecticides have been categorized into Groups 2A (cyclodiene and organochlorines, i.e., chlordane), 2B (phenylpyrazoles, i.e., fipronil), while glutamate-gated chloride channel activators, avermectins and milbemycins, are in Group 6. As of this writing, isoxazolines and *meta*-diamides have not received a category from IRAC, but these compounds will probably be assigned to a new category because of their novel actions on RDL GABA receptors.

## 5. Conclusions

In this study, the toxicity of fluralaner against *Ae. aegypti*, *An. gambiae*, and *D. melanogaster* was assessed in various exposure routes. Compared to fipronil, fluralaner had a more slowly developing toxicity, and generally a 7- to 100-fold lower potency in adult bioassays. These findings suggest that fipronil would be a better overall mosquitocide than fluralaner in the absence of resistance. The data also imply that the moderate potency, low contact toxicity, and slow action of fluralaner might preclude its use as a mosquitocide for vector control, despite its favorable mammalian selectivity and lack of cross resistance in *rdl* carrying insects.

For larval assays on *Ae. aegypti*, the concentration leading to 50% paralysis of intact larvae was 10-fold higher than that on headless larvae, so the cuticle seems to be an important factor influencing fluralaner toxicity. Compared to fluralaner, fipronil was 10- to 13-fold less potent in larval assays, which differed from the results seen in adult mosquito bioassays.

In synergism assays, DEF was found to increase fluralaner toxicity by 3.8–8 fold in feeding assays on each day of exposure, with other synergists less active. This finding suggests that carboxylamidases might be involved in the metabolism of fluralaner in mosquitoes. 

This study provides additional evidence for selectivity and lack of cross resistance of fluralaner. It was tested on mammalian GABA_A_ α_1_β_3_γ_2_ receptors, which gave an IC_50_ value larger than 30 µM. Additionally, feeding and glass contact assays of fluralaner against susceptible and *rdl* strains of *D. melanogaster* showed similar activity, consistent with the equal sensitivity of larval CNS recordings towards fluralaner in both strains. 

## Figures and Tables

**Table 1:** Larval bioassays of fluralaner on fourth-instar *Ae. aegypti* and *An. gambiae* larvae.

Species	Bioassay	LC_50_ or PC_50_, ppb; (95% CI)
*Ae. aegypti*	Intact larval toxicity in water	24 h LC_50_ 1.8 (1.4–2.2)
		48 h LC_50_ 0.5 (0.3–0.6)
	Intact larval toxicity in saline	24 h LC_50_ 2.9 (2.2–4.0)
	Intact larval toxicity in water + Triton	24 h LC_50_ 2.7 (2.1–3.6)
	Headless larval paralysis in saline	5 h PC_50_ 3.0 (1.1–6.0)
	Intact larval paralysis in saline	5 h PC_50_ 30 (26–35)
*An. gambiae*	Intact larval toxicity in water	24 h LC_50_ 0.4 (0.3–0.5)

**Table 2:** Topical bioassays of fluralaner on *Ae. aegypti* and *An. gambiae* adult females.

Compounds	Species	Time (Day)	LD_50_, ng/mg; (95% CI)
Fluralaner	*Ae. aegypti*	1	1.3 (1.0–2.3)
		2	0.50 (0.38–0.65) *
		3	0.26 (0.22–0.31) *
		4	0.21 (0.17–0.31)
		5	0.15 (0.12–0.18)
		6	0.14 (0.12–0.17)
	*An. gambiae*	1	0.29 (0.25–0.35)
		2	0.21 (0.19–0.24) *
		3	0.20 (0.18–0.23)
Fipronil	*Ae. aegypti*	1	0.062 (0.050–0.076)
		2	0.029 (0.022–0.042) *
		3	0.019 (0.015–0.023)

* Significant difference at *p* < 0.05, compared to the LD_50_ values of the preceding day.

**Table 3:** Bioassays of fluralaner and fipronil on *Ae. aegypti* adult females.

Compounds	Bioassay	Time (h)	LD_50_ or LC_50_; (95% CI)
Fluralaner	Injection	24	0.6 (0.4–0.9) ng/mg
	Glass Contact	24	13 (9–19) ng/cm^2^
		48	6 (5–9) ng/cm^2^ *
	Feeding	24	49 (34–84) ppm
		48	12 (6–20) ppm *
		72	5 (3–6) ppm
Fipronil	Glass Contact	24	10 (7–17) ng/cm^2^
		48	0.7 (0.4–1.1) ng/cm^2^ *
	Feeding	24	0.47 (0.36–0.42) ppm
		48	0.12 (0.09–0.15) ppm *

* Significant difference at *p* < 0.05, compared to the LD_50_ or LC_50_ values of the preceding day.

**Table 4:** Effects of synergists in feeding assays of fluralaner against *Ae. aegypti* adult females.

Synergist	Time (Day)	LC_50_, ppm; (95% CI)	SR ^1^
PBO	1	39 (29–58)	1.3
	2	5.5 (3.9–7.5)	2.2
	3	4.6 (2.8–8.7)	1.1
DEF	1	13 (8–24) *	3.8
	2	1.5 (1.1–2.0) *	8.0
	3	0.8 (0.5–1.2) *	6.3
DEM	1	60 (40–104)	0.8
	2	31 (21–48)	0.4
	3	12 (7–20)	0.4

^1^ Synergist ratio = LC_50_ of fluralaner (from Table 3)/LC_50_ of fluralaner + synergist; * Significant difference at *p* < 0.05, compared to data for fluralaner alone on the corresponding day (Table 3).

**Table 5:** Effects of synergists in topical assays of fluralaner against *Ae. aegypti* adult females.

Synergist	Time (Day)	LD_50_, ng/mg; (95% CI)	SR ^1^
PBO	1	0.72 (0.50–1.41)	1.8
	2	0.29 (0.22–0.37)	1.7
	3	0.19 (0.14–0.24)	1.4
	4	0.12 (0.06–0.15) *	1.8
	5	0.12 (0.06–0.15)	1.3
DEF	1	0.97 (0.77–1.38)	1.3
	2	0.36 (0.26–0.45)	1.4
	3	0.30 (0.25–0.35)	0.9
	4	0.17 (0.10–0.24)	1.2
	5	0.17 (0.10–0.24)	0.9
DEM	1	0.69 (0.55–0.97) *	1.9
	2	0.38 (0.27–0.47)	1.3
	3	0.31 (0.24–0.39)	0.8
	4	0.22 (0.15–0.29)	1.0
	5	0.22 (0.15–0.29)	0.7

^1^ Synergist ratio = LD_50_ of fluralaner (from Table 2)/LD_50_ of fluralaner + synergist; * Significant difference at *p* < 0.05, compared to the LC_50_ values of the preceding day.

**Table 6:** Glass contact and feeding bioassays of fluralaner against susceptible and resistant adult *D. melanogaster* strains.

Assay	Strain	Time (h)	LC_50_; (95% CI)	Ratio ^1^
Glass Contact (ng/cm^2^)	CS-OR	24	18 (13–27)	5.8
		48	3.1 (2.0–5.3) *	
	*rdl*-1675	24	13 (9–21)	3.9
		48	3.3 (1.7–5.3) *	
Feeding (ppm)	CS-OR	24	6.5 (3.4–12.3)	3.4
		48	1.9 (0.6–3.1) *	
	*rdl*-1675	24	7.0 (5.2–10.4)	3.9
		48	1.8 (1.0–2.7) *	

^1^ LD_50_ at 24 h/LD_50_ at 48 h; * Significant difference at *p* < 0.05, compared to the LC_50_ values of the preceding day.

## References

[1] Ozoe Y., Takeda M., Matsuda K., Ishaaya I., Horowitz A.R. (2009). γ-Aminobutyric acid receptors: A rationale for developing selective insect pest control chemicals. Biorational Control of Arthropod Pests Application and Resistance Management.

[2] IRAC Mode of Action Classification Scheme. http://www.irac-online.org/documents/moa-classification/.

[3] GABA-alpha Receptor [*Drosophila melanogaster*], Genbank: AAA28556.1. http://www.ncbi.nlm.nih.gov/protein/AAA28556.1.

[4] Rdl Mutations. http://flybase.org/reports/FBrf0213501.html.

[5] Ffrench-Constant R.H., Roush R.T. (1991). Gene mapping and cross-resistance in cyclodiene insecticide-resistant *Drosophila melanogaster* (Mg.). Genet. Res..

[6] Zhang H.G., Ffrench-Constant R.H., Jackson M.B. (1994). A unique amino acid of the *Drosophila* GABA receptor with influence on drug sensitivity by two mechanisms. J. Physiol..

[7] Casida J.E. (2015). Golden age of RyR and GABA-R diamide and isoxazoline insecticides: Common genesis, serendipity, surprises, selectivity, and safety. Chem. Res. Toxicol..

[8] Shoop W.L., Hartline E.J., Gould B.R., Waddell M.E., McDowell R.G., Kinney J.B., Lahm G.P., Long J.K., Xu M., Wagerle T. (2014). Discovery and mode of action of afoxolaner, a new isoxazoline parasiticide for dogs. Vet. Parasitol..

[9] Mita T., Kikuchi T., Mizukoshi T., Yaosaka M., Komoda M. (2005). Isoxazoline-Substituted Benzamide Compound and Noxious Organism Control Agent. WO Patent.

[10] Ozoe Y., Asahi M., Ozoe F., Nakahira K., Mita T. (2010). The antiparasitic isoxazoline a1443 is a potent blocker of insect ligand-gated chloride channels. Biochem. Biophys. Res. Comm..

[11] Gassel M., Wolf C., Noack S., Williams H., Ilg T. (2014). The novel isoxazoline ectoparasiticide fluralaner: Selective inhibition of arthropod γ-aminobutyric acid- and l-glutamate-gated chloride channels and insecticidal/acaricidal activity. Insect Biochem. Mol. Biol..

[12] Zhao C., Casida J.E. (2014). Insect γ-aminobutyric acid receptors and isoxazoline insecticides: Toxicological profiles relative to the binding sites of [^3^H]fluralaner, [^3^H]-4’-ethynyl-4-*N*-propylbicycloorthobenzoate, and [^3^H]avermectin. J. Agric. Food Chem..

[13] MRA-112 *Anopheles gambiae*, Strain G3 (*vectors*). https://www.beiresources.org/Catalog/BEIVectors/MRA-112.aspx.

[14] Islam R.M., Bloomquist J.R. (2015). A method for assessing chemically-induced paralysis in headless mosquito larvae. MethodsX.

[15] Pridgeon J.W., Becnel J.J., Clark G.G., Linthicum K.J. (2009). A high throughput screening method to identify potential pesticides for mosquito control. J. Med. Entomol..

[16] Larson N.R., Carlier P.R., Gross A.D., Islam R.M., Ma M., Sun B., Totrov M.M., Yadav R., Bloomquist J.R. (2016). Toxicology of potassium channel-directed compounds in mosquitoes. NeuroToxicology.

[17] Francis S.A.M., Taylor-Wells J., Gross A.D., Bloomquist J.R. (2017). Toxicity and physiological actions of carbonic anhydrase inhibitors to *Aedes aegypti* and *Drosophila melanogaster*. Insects.

[18] Guidelines for Testing Mosquito Adulticides for Indoor Residual Spraying and Treatment of Mosquito nets. http://apps.who.int/iris/handle/10665/69296.

[19] Bloomquist J.R., Ffrench-Constant R.H., Roush R.T. (1991). Excitation of central neurons by dieldrin and picrotoxinin in susceptible and resistant *Drosophila melanogaster* (Meigen). Pest Manag. Sci..

[20] Wood O.R., Hanrahan S., Coetzee M., Koekemoer L.L., Brooke B.D. (2010). Cuticle thickening associated with pyrethroid resistance in the major malaria vector *Anopheles funestus*. Parasit. Vectors.

[21] Hur J.H., Wu S.Y., Casida J.E. (1992). Oxidative chemistry and toxicology of *S,S,S*-tributyl phosphorotrithioate (DEF defoliant). J. Agric. Food Chem..

[22] Yu S.J., Valles S.M. (1997). Carboxylamidase activity in the fall armyworm (Lepidoptera: Noctuidae) and other Lepidoptera, Orthoptera, and Dictyoptera. J. Econ. Entomol..

[23] Yu S.J., Nguyen S.N. (1998). Purification and Characterization of Carboxylamidase from the Fall Armyworm, *Spodoptera frugiperda* (J. E. Smith). Pestic. Biochem. Physiol..

[24] Asahi M., Kobayashi M., Matsui H., Nakahira K. (2015). Differential mechanisms of action of the novel γ-aminobutyric acid receptor antagonist ectoparasiticides fluralaner (A1443) and fipronil. Pest Manag. Sci..

[25] Walther F.M., Paul A.J., Allan M.J., Roepke R.K.A., Nuernberger M.C. (2014). Safety of fluralaner, a novel systemic antiparasitic drug, in MDR1(-/-) Collies after oral administration. Parasites Vectors.

[26] Walther F.M., Allan M.J., Roepke R.K.A., Nuernberger M.C. (2014). Safety of fluralaner chewable tablets (bravecto), a novel systemic antiparasitic drug, in dogs after oral administration. Parasites Vectors.

[27] Nakao T., Banba S., Nomura M., Hirase K. (2013). Meta-diamide insecticides acting on distinct sites of RDL GABA receptor from those for conventional noncompetitive antagonists. Insect Biochem. Mol. Biol..

[28] Casida J.E., Durkin K.A. (2015). Novel GABA receptor pesticide targets. Pestic. Biochem. Physiol..

